# Self-Employment Entry Across Family Transitions: Household Capability Reallocation Over the Life Course

**DOI:** 10.1177/10422587261447788

**Published:** 2026-05-19

**Authors:** Hien Thu Tran

**Affiliations:** 1 Telfer School of Management, University of Ottawa, Ottawa, ON, Canada

**Keywords:** self-employment entry, family transitions, life course, household bargaining, capability reallocation, gendered roles, Canada

## Abstract

This study examines how major family transitions—marriage, childbirth, divorce, widowhood, and the co-occurrence of marriage and childbirth within the same observation interval—are associated with entry into self-employment as households reallocate risk, care, and income responsibilities. Using longitudinal Canadian data from the Longitudinal and International Study of Adults (2012–2020), it draws on life-course theory, household bargaining perspectives, and the capability approach to conceptualize self-employment entry as a process of contingent capability reallocation rather than simply a reflection of stable entrepreneurial preferences. The findings show that identical life events generate divergent entry responses across gendered breadwinner roles, caregiving demands, and life stage. Marriage and childbirth are positively associated with entry on average, but these associations are contingent: marriage is most strongly associated with entry among newly married secondary-earning women, while childbirth suppresses entry for primary-earning mothers and attenuates for new fathers as caregiving intensity rises. Divorce shows no uniform association: it is positively associated with childcare-expense responsibility but negatively associated with prior primary-earner status. Widowhood is associated with reduced entry, with no working-age moderation. Finally, the co-occurrence of marriage and childbirth within the same biennial interval is associated with higher entry than either transition alone.

## Introduction

Self-employment occupies an ambiguous position in entrepreneurship research. It is often framed as a route to autonomy and self-direction, with entry explained through individual motivations, human capital, and the non-pecuniary utility of being one’s own boss (Fairlie & Fossen, 2019). Yet across advanced economies, much self-employment is small-scale and closely intertwined with household constraints, especially caregiving demands and the limits of standard employment and social protection systems (OECD, 2020; Wellington, 2006). When families experience major transitions such as childbirth, partnership formation or dissolution, or spousal loss, self-employment—more than venture creation or corporate entrepreneurship—may become the most feasible way to reconcile work with care obligations and income needs (Jeon & Ostrovsky, 2016; Rouse & Kitching, 2006).

This points to a blind spot in dominant entrepreneurship theory: entry is often interpreted as an individual opportunity- or autonomy-seeking choice, even though family transitions can reorganize household roles, caregiving, and risk-bearing in ways that make self-employment a pragmatic adjustment (Carter et al., 2017; Jayawarna et al., 2014; Kanji & Vershinina, 2024). Existing frameworks may therefore understate how life-course transitions reshape the household conditions under which self-employment becomes feasible. This can obscure how household roles and disruptions generate patterned inequalities in entry alongside individual motivations. Importantly, these adjustment processes are not gender-neutral. Gendered norms and institutional arrangements shape the allocation of earning responsibility, caregiving, and labor-market flexibility, influencing whose work is treated as adjustable and whose income is expected to anchor household stability (Ahl & Marlow, 2012; Jennings & Brush, 2013; Ridgeway, 2011). Consequently, similar life-course events may generate divergent occupational responses by altering whose resources and time can be converted into a viable self-employment option (Ahl & Marlow, 2021; Thébaud, 2016).

Major family transitions—marriage, childbirth, divorce or separation, and widowhood—reconfigure the conditions under which different forms of work become feasible by reshaping income roles, caregiving demands, and household risk buffering (Browning et al., 2014; Elder et al., 2003; Raley & Sweeney, 2020). Empirical evidence shows that these life-course events are associated with heterogeneous entry into self-employment. Childbirth has been linked to increased transitions into self-employment among some parents as they adjust work and care responsibilities (Jeon & Ostrovsky, 2016). Marriage is associated with a higher likelihood of entering business ownership (Kanji & Vershinina, 2024), and divorce and widowhood have also been linked to self-employment entry, although these patterns vary across individuals and households (Özcan, 2011). While push–pull and constraint-based explanations can document such heterogeneity, they do not explain how identical life events reorganize household resources, roles, and risk-bearing to produce divergent labor-market adjustments.

We argue that resolving this puzzle requires shifting the unit of explanation from the individual entrepreneur to the household, where work choices are shaped by how resources, care, and risk are organized across members (Aldrich & Cliff, 2003; Carter et al., 2017; Welter, 2011). Integrating life-course theory with collective household bargaining and the capability approach (Chiappori, 1997; Robeyns, 2017; Sen, 1999), we conceptualize self-employment entry as a process of contingent capability reallocation. Life transitions do not simply alter household resources; they redistribute conversion capability–the capacity to turn resources, time, and support into viable work arrangements–across household members. This process is gender-structured because bargaining dynamics and role expectations shape whose labor is treated as adjustable, who absorbs care demands, and whose earnings are expected to anchor household stability. Self-employment thus emerges not only from individual autonomy-seeking, but also as a household-level adaptation to changing constraints. Under these conditions, those on the margin of adjustment—often secondary earners or primary caregivers—are more likely to shift into self-employment, while others remain anchored in wage employment even when facing the same transition (Alsos et al., 2014; Craig et al., 2012; Özcan, 2011).

This perspective is applied to specific family transitions, including the co-occurrence of marriage and childbirth within the same observation interval (Elder & Giele, 2009; Mortimer & Shanahan, 2007). We treat this co-occurrence as a form of compressed family-transition timing and examine whether it is associated with entry beyond the separate patterns linked to single events (Jayawarna et al., 2014; Jeon & Ostrovsky, 2016). When marriage and childbirth occur in close succession, the scope for incremental adjustment within wage employment narrows, increasing the likelihood that one household member reallocates capability toward self-employment.

We test these arguments using longitudinal Canadian data from the Longitudinal and International Study of Adults (LISA, 2012–2020) (Appendix A). Canada provides a theoretically revealing context because family transitions unfold within an institutional environment that offers job-protected parental leave and has pursued major childcare fee reductions and system expansion in recent years (Employment and Social Development Canada, 2024; Statistics Canada, 2023). Yet these supports do not eliminate the need for households to reallocate labor, caregiving, and risk following life events. This highlights that formal policy supports alone may not undo gendered allocations of responsibility within households, reinforcing the need to incorporate gender explicitly into a household-embedded explanation of self-employment entry (Jennings & Brush, 2013).

This study makes three contributions to the self-employment literature. First, it challenges individualistic accounts of entry by showing that self-employment can arise as a household-level adaptation to life-course transitions rather than solely from opportunity pursuit. Second, it advances contingent capability reallocation as the mechanism through which identical events generate divergent entry responses across household roles, while clarifying how gendered role allocation structures this process. Third, it shows that timing matters: the co-occurrence of marriage and childbirth within the same observation interval operates as a capability shock that elevates entry beyond the separate effects of single events. Guided by this mechanism, we ask how major family transitions and their co-occurrence reallocate household conversion capability in ways that increase or suppress self-employment entry across household roles.

Overall, our findings indicate that self-employment entry is a household-embedded outcome shaped by how families reallocate time, resources, and responsibilities, rather than a uniform expression of entrepreneurial intent. Life transitions redistribute constraints and opportunities within households, producing systematically different entry responses to the same event.

## Literature Discussion

### Contingent Capability Reallocation in Household-Embedded Entry

#### Life Events as Redistributive Transitions

Life-course research treats major family transitions—marriage, childbirth, separation/divorce, and widowhood—as turning points because they reorganize how households allocate time, manage risk, and mobilize resources across linked lives (Carr, 2018; Elder et al., 2003; Elder & Giele, 2009). Their significance lies less in changes in resource levels than in shifts in the relational organization of work and care: who is expected to provide stable earnings, who absorbs volatility, and whose time remains adjustable (Craig et al., 2012). These reallocations help explain why particular work arrangements become feasible for some household members but not others (Carter et al., 2017).

The effects of family transitions are shaped not only by event type but also by life stage and temporal proximity. The same transition may have different implications during working age—when labor-market attachment and income replacement are more pressing—than later in the life course, when planning horizons and institutional supports differ (Hutchison, 2011; Mortimer & Shanahan, 2007). Proximity also matters because transitions may co-occur rather than unfold in isolation. When obligations accumulate within the same period, households face compressed adjustment demands that are harder to absorb through gradual rebalancing within wage employment alone. This dynamic is especially relevant when marriage and childbirth co-occur, concentrating shifts in care needs and income coordination.

Such reallocations can open a window for occupational reconsideration because the routines that stabilize wage employment—fixed schedules, reliance on a single employer, and benefit-linked job continuity—become harder to sustain when care demands intensify or buffering capacity collapses. In some households, self-employment becomes comparatively viable because it offers greater temporal discretion, facilitates income smoothing, or allows one member to absorb uncertainty without requiring the entire household to withdraw from paid work. In others, the same transition tightens constraints by reducing slack and increasing exposure to downside risk, thereby reinforcing attachment to wage employment. Occupational adjustment is therefore not reducible to individual preferences or human capital; it depends on whether a transition reallocates risk, time, and dependency in ways that expand or compress the capability to pursue self-employment (Carter et al., 2017; Wellington, 2006).

Entrepreneurship scholarship increasingly supports this household-embedded view of entry. Work choices are negotiated within households and shaped by spousal earnings, the division of care, and informal insurance through partner support (Alsos et al., 2014). Evidence also shows that identical family circumstances can enable entry for one household member while constraining another, depending on whose work is more adjustable and how caregiving responsibilities are distributed within the couple (Kanji & Vershinina, 2024; Naldi et al., 2021). In this sense, family transitions are not simply “triggers” of opportunity or constraint; they can reallocate conversion capability—the household- and institution-shaped capacity to translate resources, time, and support into feasible work arrangements. This, in turn, reshapes who can plausibly adjust their occupational pathway toward self-employment.

#### Household Bargaining and Institutional Context as the Relational Foundations

Prior self-employment research has often interpreted family transitions through constraint-based accounts that emphasize intensified caregiving demands and limited schedule control, or through push-pull logics in which resource loss pushes necessity entry while autonomy and flexibility pull some individuals toward self-employment (Ekinsmyth, 2013; Millán et al., 2014; Parker, 2018). These perspectives identify important correlates, but they typically treat entry as an individual response to resources or preferences. This is insufficient to explain why identical life events generate divergent entry responses across households. Addressing this requires treating the household as an allocating institution in which bargaining positions and role arrangements shape who absorbs risk and care demands, and whether self-employment becomes feasible (Carter et al., 2017; Jennings & Brush, 2013).

Household bargaining theory offers a relational mechanism for understanding self-employment entry by conceptualizing labor supply, caregiving, and resource use as negotiated outcomes rather than isolated individual decisions (Bittman et al., 2003; Chiappori, 1992). Household members differ in fallback options and claims on shared resources, while role expectations structure responsibilities for care and income provision, shaping whose labor is adjustable and whose earnings anchor household security (Anderson & Eswaran, 2009; Lundberg & Pollak, 1996). This framing aligns with self-employment research showing that partner employment, income pooling, and caregiving allocation condition the feasibility and timing of labor-market transitions, including entry into self-employment (Carter et al., 2017).

However, much applied empirical work operationalizes household bargaining in relatively static terms, implicitly treating bargaining positions and divisions of care as stable over short horizons (Chiappori & Mazzocco, 2017). This assumption conflicts directly with the nature of life-course transitions. Childbirth introduces non-substitutable care that can reshape whose time is elastic; separation dissolves income pooling and reorganizes dependency; and widowhood removes spousal support while exposing care needs previously managed within the couple (DiGiacomo et al., 2013; Keene & Prokos, 2008; Le Bourdais et al., 2016). These events do not merely prompt negotiation; they alter the conditions under which negotiation occurs. Treating bargaining as static therefore obscures the dynamic reallocations of responsibility and risk that follow life events and shape whether self-employment becomes feasible or foreclosed.

Institutional context further conditions how households absorb or externalize family-transition shocks. Social policy and labor-market institutions shape how childcare, retirement security, and income protection are distributed between the state and families, thereby shaping whether adjustment occurs through market work, intra-household risk absorption, or reduced labor supply (Gornick & Meyers, 2003; Leitner, 2003). Household financial arrangements, especially control over income and access to independent resources, also affect how costs and adjustments are allocated within couples (Bennett & Sung, 2013). Together, these considerations motivate a framework centered not only on negotiation but also on the post-transition reallocation of feasibility for self-employment.

#### Capability Reallocation as the Mechanism for Differentiated Responses

To integrate these insights, this study conceptualizes self-employment entry as a process of contingent capability reallocation. In the capability approach, labor-market decisions depend not only on access to resources, but on the real opportunities individuals have to convert those resources into feasible work arrangements under specific personal and relational conditions (Robeyns, 2017; Sen, 1999). This framework distinguishes between resources or means (e.g. income, assets, benefits, partner support), the conversion factors that shape how those resources can be used (e.g. health, skills, caregiving demands, and intra-household role arrangements), and the resulting feasible work options (Comim et al., 2008). This matters because households may hold similar resources yet differ substantially in whether those resources can be translated into viable self-employment. Since conversion factors are relational, the same household resources or financial slack may expand one partner’s feasible options while tightening constraints for the other. Capability, in this sense, lies not in possessing resources but in being able to convert them into genuine occupational choice (Robeyns, 2017; Sen, 1999).

Life events rarely alter these conversion conditions uniformly. More often, they reshape how resources and time can be turned into feasible work options. Family transitions can reconfigure income pooling and risk buffering, redistribute care obligations, and shift expectations about whose labor should remain stable versus adjustable. As a result, the same transition may expand one household member’s feasible options while compressing another’s, creating within-household differences in the viability of self-employment even when observable resources appear similar (Craig et al., 2012).

We argue that capability reallocation is conditioned by three factors that shape whether flexibility becomes feasible, necessary, or precluded. First, earning role determines a household’s tolerance for income volatility, because bargaining positions and role expectations help organize whose earnings anchor household stability (Bertrand et al., 2015; Lundberg & Pollak, 1996). Second, caregiving intensity shapes time elasticity and the value of schedule control relative to income certainty, as care demands tighten time constraints and reconfigure the division of labor around family transitions (Ekinsmyth, 2013; Yavorsky et al., 2015). Third, remaining working-life horizon shapes whether the upfront costs and uncertainty of entry can plausibly be recovered over time, and whether delayed or uncertain returns make self-employment less attractive than immediate wage income (Azoulay et al., 2020; Lévesque & Minniti, 2006).

Gender is theorized in gender-and-work and feminist scholarship as a socially institutionalized structure that organizes role expectations and constraints, rather than as an identity-based preference or fixed essence (Moen, 2016). In this study, gender operates as a cross-cutting conversion condition that shapes how couples sort into breadwinner and secondary-earner positions, whose labor is treated as adjustable, and how caregiving responsibilities are allocated when family circumstances change (Ahl & Marlow, 2012; Ridgeway, 2011). Its effects are not expected to be uniform across all life events. Gender is most likely to matter when a transition activates and reorders within-household role expectations around specialization, caregiving, and earning responsibility, because these processes shape whose time and resources can be converted into a viable self-employment option (Yavorsky et al., 2015). Accordingly, we theorize that gender is most salient in unification transitions, particularly marriage and childbirth, when couples renegotiate labor adjustability, caregiving, and income anchoring. By contrast, dissolution transitions, especially divorce and widowhood, operate primarily through the loss of pooling and shared buffering, reducing conversion capability through diminished insurance and support and constraining both women and men once post-transition roles and resources are taken into account (Chiappori & Mazzocco, 2017).

This framework advances self-employment research by moving beyond resource-based and push–pull logics that assume linear effects of advantage or scarcity. Instead, self-employment becomes feasible not simply when resources change, but when life events reallocate the capacity to convert resources and time into a viable work arrangement within the household. Capability reallocation therefore links family transitions to entry through conditional, context-dependent pathways shaped by earning roles, caregiving demands, and time horizon (Ekinsmyth, 2013; Lévesque & Minniti, 2006; Lundberg & Pollak, 1996).

### Hypotheses—Event-Specific Capability Reallocation

To derive testable predictions, we move from the general mechanism of contingent capability reallocation to event-specific expectations. Marriage, childbirth, separation/divorce, and widowhood are treated as distinct life-course shocks that reorganize household buffering, time elasticity, and role configurations, thereby reshaping who can credibly convert resources and time into self-employment. On this basis, we develop hypotheses that differentiate effects by earning role, caregiving intensity, and remaining working-life horizon.

#### Marriage

Marriage is a major life-course turning point because it merges two previously separate economic trajectories into a jointly organized household in which labor supply, risk management, and caregiving are negotiated across linked lives (Chiappori & Mazzocco, 2017; Elder, 1994; Killewald & Gough, 2013). This matters for self-employment because entry often entails uncertainty in earnings, hours, and benefits. Whether an individual can act on entrepreneurial intentions therefore depends not only on personal resources, but also on the household’s capacity to absorb volatility while reorganizing work and care.

From a capability reallocation perspective, marriage can expand the feasibility of entry by strengthening household-level buffering and support. Pooling earnings and consumption can reduce exposure to short-run income shocks and ease liquidity constraints, lowering the effective risk of entry even when the individual’s own resources are unchanged (Özcan, 2011). Marriage can also increase relational and practical support that helps households tolerate early-stage instability and workload pressures, which are often decisive during start-up (Powell & Eddleston, 2013; Xu et al., 2020). In addition, it may expand access to family social capital and spousal know-how, reducing informational and practical barriers to entry (Edelman et al., 2016).

However, marriage does not simply pool resources; it also reorders how those resources can be converted into occupational choices. Because marriage is socially embedded, it activates institutionalized expectations about specialization in paid and unpaid work, shaping whose labor is treated as adjustable and whose earnings are expected to anchor household stability (Killewald & Gough, 2013). In this framework, unification transitions make gendered conversion structures especially salient because they reconfigure perceived earning responsibility and the legitimacy of flexibility-seeking choices at the moment couples renegotiate roles.

This logic yields a sharper prediction than the simple claim that secondary earners benefit. Even among individuals in similar economic positions, women and men may face different normative constraints on how marriage-related buffering can be used. For secondary-earning women, marriage may convert additional household insurance into discretionary slack and a socially legible route into flexible work, making self-employment more feasible (Bari et al., 2021; Kanji & Vershinina, 2024). For secondary-earning men, by contrast, breadwinner norms and expectations of job stability may remain binding even when their income share is secondary, limiting the extent to which marriage-related resources translate into entrepreneurial feasibility (Bertrand et al., 2015; Gonalons-Pons & Gangl, 2021). Thus, the same increase in marriage-related buffering may expand conversion capability for secondary-earning women while leaving comparable men more tightly bound to norms of stable wage work (Bjuggren & Henrekson, 2022). Marriage is therefore expected to shape self-employment entry in role-contingent and gender-structured ways. Accordingly, we propose:

**H1a.** Entering marriage is associated with a higher likelihood of entering self-employment.
**H1b.** This positive association is stronger for secondary-earning women.

#### Childbirth

Childbirth is a consequential life-course transition because it increases non-substitutable caregiving demands, compresses time availability, and raises household dependency (Bianchi et al., 2012; Craig & Mullan, 2011). In life-course terms, the birth of a child is not an individual shock but a linked-lives reorganization in which paid work, unpaid work, and risk management are recalibrated across the household (Aguilar-Gomez et al., 2026). Empirical evidence shows that childcare remains unevenly allocated even in dual-earner households: mothers continue to absorb more routine and coordinating care, while fathers’ adjustments, though real, are typically smaller and more contingent on work context and policy settings (Drew & Humbert, 2012; Yavorsky et al., 2015). This unequal redistribution creates a persistent care-intensity penalty that shapes labor-market options and the feasibility of self-employment.

From a capability reallocation perspective, childbirth can either constrain or enable self-employment entry because it reshapes the conversion conditions under which time and resources can be turned into a viable occupational move. Early-stage self-employment often requires irregular effort, administrative overhead, and tolerance for income volatility, all of which can clash with intensified caregiving and fragmented time. Yet where households have sufficient buffers and role arrangements that make flexibility usable, self-employment may become an actionable adjustment, especially when it is interpreted as compatible with caregiving-intensive parenting (Budig & Hodges, 2010).

For secondary-earning mothers, childbirth can make self-employment more feasible when the household can buffer early income volatility through a partner’s earnings, savings, or other resources, and when self-employment is treated as an acceptable way to remain attached to the labor market while accommodating intensified care demands. This is consistent with Canadian evidence linking new motherhood to movement from wage employment into self-employment (Jeon & Ostrovsky, 2016). At the same time, this reconciliation pathway is contingent rather than universal. Where public supports are limited, childcare is difficult to secure, or work schedules are rigid, self-employment may function as a practical coping strategy rather than a preferred option, and may not draw mothers away from wage employment in every setting (Bari et al., 2021; Kanji & Vershinina, 2024; Matysiak & Mynarska, 2020).

The prediction is different for sole- and primary-earning mothers. When the mother’s earnings anchor household stability, childbirth raises the cost of leaving stable wage employment and intensifies exposure to downside risk at precisely the point when expenses and time constraints increase. In capability terms, the key constraint is not reduced aspiration, but compressed conversion capacity: heightened financial exposure, time scarcity, and career-penalty dynamics reduce the feasibility of moving into uncertain work. This is consistent with evidence on the long-run career costs of children and persistent post-birth gender inequality in earnings and labor-market trajectories (Adda et al., 2017; England et al., 2016; Kleven et al., 2019).

For fathers, childbirth often reinforces provider-centered conversion conditions. Even as norms of involved fathering strengthen, the arrival of a child can intensify expectations that men sustain earnings and remain continuously attached to paid work. These pressures are maintained not only through breadwinner norms, but also through workplaces organized around an “ideal worker” standard that penalizes visible claims on time for caregiving. In capability terms, childbirth can therefore preserve or even tighten men’s attachment to wage employment by raising the perceived cost of work arrangements that signal reduced availability (Rudman & Mescher, 2013; Thébaud, 2010). Fathers’ responses are not uniform, however. When caregiving intensity rises—for example, with multiple preschool children, limited childcare access, or nonstandard schedules—their constraints can begin to resemble those more often documented for mothers (Altintas & Sullivan, 2017; Chesley, 2011; McGill, 2014). Because ideal-worker norms continue to valorize continuous availability and remain historically masculinized (Kossek et al., 2021), self-employment may amplify rather than relieve capability strain for fathers with substantial caregiving responsibilities by intensifying long-hours demands and work-family conflict (Annink et al., 2016; Dinh et al., 2021).

One implication of capability reallocation is that constraints bind most tightly when post-birth role demands conflict with normative role expectations. When childbirth reinforces conventional allocations—mothers absorbing most care and fathers prioritizing continuous earnings—self-employment may appear more feasible for some women as a care-compatible adjustment, while men remain normatively channeled toward stable wage work. When roles are counter-normative—women as sole or primary earners and men as intensive caregivers—conversion capability is more likely to compress, as financial exposure, time rigidity, and stigma around reduced availability jointly reduce the feasibility of entering volatile and time-demanding work (Chung & van der Lippe, 2020; McGill, 2014; Rudman & Mescher, 2013). Accordingly, we hypothesize:

**H2a**. Childbirth is associated with a higher likelihood of entering self-employment.
**H2b.**This positive association is weaker for women in sole- and primary-earner positions.
**H2c**. This positive association is weaker for men experiencing high caregiving intensity.

#### Divorce

Divorce is a disruptive life-course transition because it dissolves household pooling and reorganizes previously shared arrangements for income, time, and caregiving into more individualized obligations (Elder et al., 2003). In life-course terms, the end of “linked lives” matters because the household unit that coordinated labor supply and absorbed risk is reconfigured under new financial, temporal, and emotional constraints (Leopold, 2018; Tamborini et al., 2015). In Canada, divorce and separation are associated with substantial income losses and post-separation adjustments shaped by employment and childcare arrangements, underscoring that the consequences of dissolution are already significant even before occupational choice is considered (Le Bourdais et al., 2016). Post-dissolution caregiving arrangements are also heterogeneous: some children live primarily with one parent, while others move between shared-care arrangements across households. In the Canadian context, mothers remain more likely than fathers to be the child’s primary residential parent after separation or divorce (Statistics Canada, 2021). As a result, divorce does not generate a uniform redistribution of care, time, and financial obligations across former partners, and its implications for self-employment feasibility are correspondingly uneven.

From a capability reallocation perspective, the dominant mechanism is the collapse of conversion capability: the loss of pooling, coordination, and mutual insurance reduces the ability to turn resources, time, and support into feasible work arrangements. This implies heterogeneous effects on self-employment, conditioned by caregiving burdens and pre-divorce earning roles. For some individuals, dissolution may raise the value of autonomy and control over work. For others, the loss of shared buffering compresses their capability to tolerate earnings volatility and invest time in a start-up phase. Consistent with this asymmetry, research on partner separation among the self-employed finds increased movement out of self-employment following separation, suggesting that dissolution often tightens constraints rather than opens opportunity (van Loon et al., 2020). Macro evidence also links divorce dynamics and self-employment rates, but it should be interpreted cautiously because it does not identify within-person entry responses to divorce (Saridakis et al., 2018).

Caregiving responsibilities are central to this heterogeneity. When divorce coincides with intensified childcare obligations, especially when individuals assume direct responsibility for childcare expenses, conversion capability may be compressed rather than expanded. Childcare costs reduce disposable income and tighten liquidity, limiting the capacity to absorb the short-term earnings volatility that often accompanies self-employment entry (Casarico et al., 2023). Under such conditions, wage employment may be comparatively attractive because it offers predictable earnings and institutional risk protection that self-employment cannot easily replicate (Hansson et al., 2024; OECD, 2022). When caregiving demands are lower or can be reorganized, however, dissolution may expand discretion over time use and decision-making, making autonomy-seeking work adjustments more feasible. Because autonomy and control remain central non-pecuniary motives for self-employment even under income uncertainty (Benz & Frey, 2008), and divorce can reorient time use and work priorities for some individuals (Wanberg et al., 2023), self-employment may become a plausible route to restore autonomy and rebuild earnings capacity when constraints permit (Almog & Herbst-Debby, 2025).

Pre-divorce earning roles further condition post-divorce feasibility. Bargaining approaches emphasize that fallback positions and resource control shape post-transition options (Bittman et al., 2003; Lundberg & Pollak, 1996). Earning-role configurations within marriage also leave legacies for post-divorce trajectories, shaping who can absorb income risk and reallocate time toward self-employment after dissolution (Brüggmann & Kreyenfeld, 2023). Primary earners, in particular, may be better positioned to pursue self-employment because they are more likely to retain stronger labor-market attachment, access to credit, and earnings capacity that can be redeployed when post-divorce constraints loosen rather than intensify (Özcan, 2011). Accordingly, we propose the following hypotheses:

**H3a**. The association between divorce and self-employment entry is heterogeneous and depends on post-dissolution capability conditions.
**H3b**. This association is weaker when individuals assume responsibility for childcare expenses.
**H3c**. This association is stronger for individuals who were sole or primary earners prior to divorce.

#### Widowhood

Widowhood is an abrupt life-course disruption because it removes the relational arrangement through which partners jointly coordinate income generation, household production, and day-to-day support. Its immediate consequence is the loss of pooling and mutual substitution, often triggering substantial economic adjustment and heightening the risk of financial insecurity for the survivor (Carr & Utz, 2020). Evidence also shows that widowhood is commonly accompanied by declines in household wealth, particularly around the transition itself, thereby reducing the financial slack that helps absorb income volatility (Hurd & Wise, 1989; Kapelle & Van Winkle, 2024).

From a capability reallocation perspective, the implications for self-employment entry depend strongly on timing. When spousal loss occurs during working life, survivors must replace income while reorganizing household management under new constraints. Administrative evidence suggests that surviving spouses may increase labor supply following fatal shocks, consistent with income-replacement pressure as an immediate post-loss adjustment mechanism (Fadlon & Nielsen, 2021). Under these conditions, self-employment becomes a plausible adjustment only when survivors retain residual capability—marketable skills, viable networks, and some financial buffer—and when it offers more workable control over time than available wage options.

When widowhood occurs closer to retirement age, capability constraints are more likely to bind. Shorter planning horizons reduce the expected returns to business investment, financial vulnerability lowers tolerance for earnings volatility, and health limitations can restrict capacity for time-intensive market work. Under these conditions, entry into self-employment becomes less feasible as a post-bereavement adjustment and helps explain why later-life widowhood is more often associated with labor-force withdrawal than with new work reconfiguration (Carr & Utz, 2020; Kapelle & Van Winkle, 2024). Support substitution further conditions post-bereavement capability: assistance from adult children, extended kin, neighbors, and friends can partly offset the loss of spousal support, but access to such support is uneven and may not endure (Kalmijn, 2019; Klaus, 2021).

Overall, widowhood is expected to reduce the feasibility of self-employment entry because it removes pooling and lowers tolerance for risk. However, when widowhood occurs during working age, this negative association should be weaker when survivors retain residual capability and can substitute support. Accordingly, we propose:

**H4a**. Widowhood is associated with a lower likelihood of entering self-employment.
**H4b**. This negative association is weaker when widowhood occurs during working age.

### Rapid Family Formation and the Co-occurrence of Marriage and Childbirth

Life-course theory emphasizes that family transitions are shaped not only by event type but also by timing and proximity. When major transitions occur close together, they concentrate adjustment demands and generate consequences that single-event accounts may miss (Elder & Giele, 2009). Here, co-occurrence refers to marriage and childbirth taking place within the same biennial interval, regardless of sequence. In such cases, households must absorb marriage formation or consolidation at the same time that intensive caregiving begins. This compresses the window for renegotiating the division of labor, recalibrating work commitments, and building buffers before parental demands intensify (Yavorsky et al., 2015).

Family embeddedness research helps explain why this matters for occupational choice. Entrepreneurial and labor-supply decisions are often made as part of a household strategy that reallocates time, income roles, and risk-bearing capacity in response to changing family demands (Aldrich & Cliff, 2003; Alsos et al., 2014). When marriage and childbirth co-occur, the scope for incremental adaptation narrows: households have less room to respond through gradual adjustments within wage employment, such as reduced hours, delayed role renegotiation, or career rotation. Under these conditions, self-employment may become an adaptive option because it can reorganize market work around intensified caregiving demands, especially for the household member whose labor is more adjustable or whose opportunity cost of leaving wage employment is lower (Jayawarna et al., 2021).

Household bargaining theory clarifies the mechanism behind this compressed reallocation. When two major transitions co-occur, bargaining over paid work, unpaid work, and risk absorption takes place under time pressure, while fallback positions and perceived obligations are renegotiated simultaneously (Chiappori & Mazzocco, 2017; Lundberg & Pollak, 1996). This compression increases the value of flexibility precisely when buffering capacity is most strained: caregiving needs rise quickly, but households have had little time to stabilize finances, reorganize schedules, or build institutional supports. In capability terms, co-occurrence operates as a concentrated capability shock, simultaneously increasing the need for flexibility and reducing the scope for gradual adjustment. As a result, self-employment entry becomes more likely than would be expected from marriage or childbirth in isolation. Thus, we propose:

**H5**. Experiencing marriage and childbirth within the same biennial interval is associated with a higher likelihood of entering self-employment than experiencing either transition alone.

## Data and Variables

### Data and Sample

Canadian scholarship on self-employment and life-course transitions remains relatively limited, despite Canada’s distinctive institutional context. Maternity and parental benefits are provided through Employment Insurance (EI) for eligible employees, while access for the self-employed depends on voluntary registration for EI special benefits, creating uneven coverage across workers and households (Government of Canada, 2023, 2025a). At the same time, Canada has expanded childcare affordability reforms and introduced sustained policy support for women’s entrepreneurship (Government of Canada, 2025b; Innovation, Science and Economic Development Canada, 2025). Yet women remain underrepresented in business ownership and self-employment (Statistics Canada, 2025; Uppal, 2023). Taken together, these features—including uneven benefit coverage, evolving childcare supports, and an individual rather than joint income tax structure—make Canada a useful setting for examining how households adjust labor-market behavior around family transitions (Government of Canada, 2025b). This study therefore provides Canada-specific evidence to a broader literature on gendered patterns of entrepreneurial activity in high-income economies (GEM, 2024).

We use the LISA, a Statistics Canada panel that follows a nationally representative sample of individuals and households biennially and collects detailed information on employment, income, family structure, and major life events. The analysis draws on five waves (2012–2020) and models transitions into self-employment using a person-interval design. Respondents contribute intervals in which they are not self-employed at the start of the wave and are therefore at risk of entry. The dependent variable indicates whether an individual enters self-employment between consecutive waves (t−1 → t). We also include a time-varying indicator for prior self-employment experience among those not currently self-employed, allowing prior exposure to be accounted for when estimating the association between family transitions and subsequent entry. Across the analytic sample, we observe more than 2,500 transitions into self-employment and the following life-course events: marriage (1,699), childbirth (3,367), divorce (874), and widowhood (464).^1^

Although measures rely on self-reports, the design mitigates contemporaneous reporting concerns because predictors are lagged and transitions are defined between biennial waves, reducing common-source inflation within the same wave.

### Variables

#### Self-Employment Entry

Self-employment entry is coded 1 when a respondent is not self-employed at t−1 (i.e. wage-employed, unemployed, or inactive) and reports self-employment at *t*; otherwise, it is coded 0 for at-risk intervals. Individuals contribute person-time only for consecutive interview pairs in which they are observed and are not self-employed at t−1 . Respondents who are continuously self-employed across all observed waves are excluded from the risk set. Individuals are right-censored at their final observation if no entry occurs.

#### Life-Course Events

Binary indicators capture whether each major life event occurs between consecutive waves. *Marriage* equals 1 when an individual enters a legal marriage or a new common-law partnership. *Childbirth* equals 1 when an individual reports the birth of or arrival of a child since the previous interview. *Divorce/separation* equals 1 when marital status shifts from married or common-law to separated or divorced, thereby capturing dissolution in both legal marriages and common-law partnerships. *Widowhood* equals 1 when a spouse or partner dies and marital status changes to widowed.

#### Household Earning Roles

Earning roles are measured using breadwinner status, defined as the respondent’s share of total household after-tax income. At each wave, individuals are classified into four mutually exclusive categories: *sole breadwinner* (100% of household income), *primary breadwinner* (>50% and <100%), *co-breadwinner* (45%–55%), and *secondary breadwinner* (<45%), with co-breadwinner as the reference category. This operationalization reflects household bargaining logic in which relative earnings shape decision authority and the allocation of paid and unpaid work within families (Killewald & Gough, 2013; Lundberg & Pollak, 1996).

#### Care Constraint and Caregiving Intensity

*Care constraints* are captured with a binary indicator equal to 1 when respondents report paying childcare expenses in the current wave. *Caregiving intensity* is measured using time-varying counts of preschool-aged children (under 6) and school-aged children (ages 6–15). Distinguishing these age groups reflects evidence that care demands are highest before school entry and remain consequential through the school years for employment adjustment.

#### Demographic Controls

*Age* (in years) and a *working-age* indicator (1 = ages 15–65) account for life-course variation. *Gender* (1 = female), *marital status* (married/common-law), *rural residence*, and *immigrant* status (landed immigrant) capture sociodemographic context. *Education* is measured with categorical indicators (less than high school, vocational or college, bachelor’s degree, master’s, or PhD). *Health* is self-rated on a four-point scale from poor to excellent. *Job tenure* is measured as years in the current or most recent job. *Prior self-employment experience* is included as a time-varying indicator distinguishing first-time from repeat entrants.

#### Industry Controls

Industry of employment is measured using major NAICS sectors (e.g. agriculture, mining, utilities, construction, manufacturing, trade, transportation, finance, professional services, public administration). Sector fixed effects account for systematic differences in baseline entry rates across industries.

#### Provincial Controls

Province of residence is captured with binary indicators for Alberta, British Columbia, Manitoba, Nova Scotia, Ontario, Quebec, Saskatchewan, and New Brunswick. Prince Edward Island and Newfoundland and Labrador are combined because of small sample sizes.

#### Year Effects

Wave dummies capture common macroeconomic conditions and policy changes over time.

## Methodology

To test the hypotheses, we estimate discrete-time event-history models using logistic regression, which is appropriate when transitions are observed in fixed intervals rather than at exact dates (Allison, 1984; Beck et al., 1998). The outcome is self-employment entry between consecutive biennial LISA waves. Each respondent contributes person-interval observations only in periods in which they are observed in two consecutive waves and are not self-employed at the start of the interval (t−1) . Individuals are right-censored at their final observed interval if no entry occurs during the observation window.

We estimate the conditional association between life-course transitions and self-employment entry as:

(1) $$\mathrm{logit}\left(P(Y_{\mathrm{it}}=1)\right)=\alpha +\lambda_{t}+\beta \mathrm{LifeEvent}s_{\mathrm{it}}+\gamma \mathrm{CapabilityCondition}s_{\mathrm{it}}+\delta X_{\mathrm{it}}$$

where $Y_{i,t}=1$ indicates entry into self-employment between waves; $\lambda_{t}$ denotes wave fixed effects; $\mathrm{LifeEvent}s_{\mathrm{it}}$ includes indicators for marriage, childbirth, divorce, widowhood, and the marriage-childbirth co-occurrence; $\mathrm{CapabilityCondition}s_{i,t}$ captures the hypothesized contingencies through interactions between life events and (a) household earning roles, (b) caregiving constraints and intensity, and (c) working-age status; and $X_{i,t}$ includes time-varying and time-invariant controls, including demographic, socioeconomic, industry, and provincial characteristics. Standard errors are clustered at the individual level to account for repeated observations per respondent.

### Mapping Hypotheses to Model Terms and Interpretation

Because the study is observational, coefficients are interpreted as conditional associations rather than causal effects. The hypotheses are evaluated through the main life-event indicators and their theoretically specified interactions. The marriage hypothesis is assessed through the main marriage indicator and an interaction capturing newly married women in secondary-earner positions. The childbirth hypotheses are assessed through the main childbirth indicator and interactions capturing new mothers in sole- or primary-earner positions and new fathers under higher caregiving intensity. The divorce hypotheses are assessed through the main divorce indicator and interactions with childcare-expense responsibility and prior sole- or primary-earner status. The widowhood hypotheses are assessed through the main widowhood indicator and its interaction with working-age status. The co-occurrence hypothesis is assessed through the joint occurrence of marriage and childbirth within the same biennial interval. Support is evaluated by whether the estimated associations are directionally consistent with the theoretical expectations.

### Sensitivity Analyses and Scope of Inference

Unobserved traits and unmeasured shocks may still confound the estimated associations. To assess robustness, we re-estimate the models using a mixed-effects (random-intercept) logistic specification, use covariates lagged to the start of each interval, and include lead indicators of life events to test for anticipatory behavior or reverse timing. These checks do not turn the design into a causal test, but the substantive patterns remain consistent with the main findings. The results are therefore interpreted as conditional associations.

## Estimation Results

Tables 1 and 2 report descriptive statistics and pairwise correlations for the key variables. The mean age is 46.86; 65% of respondents are married or in a common-law union, 18% are immigrants, and 26% live in rural areas. Correlations are generally modest, with the largest being the expected mechanical relationship between age and the working-age indicator (r=0.65) . VIF values remain below the conventional threshold of 10, and because age and working-age are not entered together in the same specifications, multicollinearity is unlikely to affect the regression estimates.

**Table 1.** Variable Definitions and Descriptive statistics.

Variables	Definition	Mean	SD
Self-employment entry	Dummy equals 1 if the respondent is not self-employed at t−1 (wage-employed, unemployed, or inactive) and reports self-employment at *t*; 0 otherwise.	0.02	0.14
Life events			
Marriage	Dummy equals 1 if the respondent reports being married in the current survey but was not in the previous one; 0 otherwise.	0.03	0.16
Childbirth	Dummy equals 1 if the respondent answers “yes” to the question “Since the last survey, did you give birth to/father a child?”; 0 otherwise.	0.04	0.19
Divorce	Dummy equals 1 if the respondent reports being divorced/separated in the current survey but was not in the previous one; 0 otherwise.	0.01	0.11
Widowhood	Dummy equals 1 if the respondent answers “yes” to the question “Since the last survey, did you experience the death of a spouse or partner?”; 0 otherwise.	0.01	0.08
Demographics			
Age	Age in the year of interview	46.86	18.36
Working age	Dummy equals 1 if the respondent is between 15 and 65 years old; 0 otherwise.	0.84	0.37
Gender	Gender of the respondent: female 1; male 0	0.47	0.50
Married	Dummy equals 1 if the respondent is married or has a common law partner; 0 otherwise.	0.65	0.48
Immigrant	Dummy equals 1 if the respondent is a landed immigrant in Canada; 0 otherwise.	0.18	0.38
Rural	Dummy equals 1 if the respondent is living in rural area; 0 otherwise.	0.26	0.44
Education	Categorical variable coded as: 0 = less than high school; 1 = vocational/college; 2 = bachelor’s; 3 = master’s/PhD	0.96	0.97
Health condition	Self-evaluated health self-assessment scale, from 0 (poor) to 3 (excellent)	2.09	0.63
Job tenure	Number of years that the respondent has worked in the last or current job.	6.24	9.30
Self-employment exp	Dummy equals 1 if the respondent is a returned self-employed; 0 otherwise	0.01	0.11
Household dynamics			
Sole breadwinner	Dummy equals 1 if the respondent’s share of household net income = 100%; 0 otherwise	0.18	0.38
Primary breadwinner	Dummy equals 1 if the respondent’s share of household net income >50% & <100%; 0 otherwise	0.30	0.46
Co-breadwinner	Dummy equals 1 if the respondent’s share of household net income ≥45% & ≤50%; 0 otherwise	0.28	0.45
Secondary breadwinner	Dummy equals 1 if the respondent’s share of household net income <45%; 0 otherwise	0.24	0.43
Caregiving responsibility			
Childcare expenses	Dummy equals 1 if the respondent pays for all childcare expenses; 0 otherwise	0.07	0.25
Caregiving intensity	Number of preschool-aged children in the household (less than 6 years old)	0.13	0.43
	Number of school-aged children in the household (6–15 years old)	0.30	0.71

*Note.* All variables are extracted from the Longitudinal and International Study of Adults survey in Canada in the period 2012 to 2020. Minima and maxima are omitted in accordance with Statistics Canada disclosure control (vetting) requirements.

**Table 2.** Pairwise Correlation Matrix.

Var.	(1)	(2)	(3)	(4)	(5)	(6)	(7)	(8)	(9)	(10)	(11)	(12)	(13)	(14)	(15)	(16)	(17)	(18)	(19)	(20)	(21)	(22)
(1)	1.00																					
(2)	0.01*	1.00																				
(3)	0.02*	0.06*	1.00																			
(4)	0.01	−0.02*	−0.02*	1.00																		
(5)	−0.00	−0.01*	−0.02*	0.41*	1.00																	
(6)	−0.01	−0.14*	−0.16*	0.07*	0.10*	1.00																
(7)	0.03*	0.06*	0.09*	−0.05*	−0.10*	−0.65*	1.00															
(8)	0.02*	−0.00	0.00	−0.02*	−0.03*	−0.02*	−0.00	1.00														
(9)	0.03*	0.12*	0.12*	−0.16*	−0.12*	0.34*	−0.02*	0.02*	1.00													
(10)	0.01	−0.03*	−0.02*	−0.00	0.00	0.08*	−0.03*	0.01*	0.07*	1.00												
(11)	0.04*	0.04*	0.06*	−0.01*	−0.03*	0.06*	0.08*	−0.02*	0.22*	−0.11*	1.00											
(12)	0.02*	−0.03*	−0.04*	−0.00	−0.02*	0.13*	0.17*	0.05*	0.21*	0.04*	0.15*	1.00										
(13)	0.02*	0.02*	0.05*	−0.02*	−0.02*	−0.21*	0.14*	−0.00	−0.02*	−0.03*	0.12*	0.07*	1.00									
(14)	0.01*	−0.03*	0.01	−0.00	−0.00	0.07*	−0.03*	−0.01*	0.07*	−0.18*	0.11*	−0.03*	−0.02*	1.00								
(15)	0.03*	0.01*	0.60*	−0.02*	−0.02*	−0.20*	0.13*	−0.01*	0.18*	−0.03*	0.09*	−0.03*	0.05*	0.02*	1.00							
(16)	0.03*	−0.03*	0.01	0.00	−0.02*	−0.09*	0.18*	−0.02*	0.23*	−0.00	0.12*	0.09*	0.04*	0.07*	0.11*	1.00						
(17)	0.32*	−0.01	0.00	−0.00	−0.00	0.02*	−0.00	0.01*	0.03*	0.01*	0.02*	0.04*	0.01*	0.01	0.01	0.02*	1.00					
(18)	0.01*	−0.01	0.21*	0.01	−0.02*	−0.15*	0.12*	−0.04*	0.13*	−0.02*	0.14*	0.02*	0.05*	−0.00	0.44*	0.25*	−0.00	1.00				
(19)	−0.00	−0.02*	−0.06*	0.18*	0.13*	0.10*	−0.14*	−0.03*	−0.54*	−0.06*	−0.02*	−0.08*	−0.07*	−0.04*	−0.09*	−0.13*	−0.01	−0.05*	1.00			
(20)	−0.00	0.04*	0.06*	−0.04*	−0.03*	0.16*	−0.04*	0.25*	0.36*	0.02*	0.17*	0.18*	0.01	0.01*	0.10*	0.12*	0.00	0.07*	−0.31*	1.00		
(21)	0.00	−0.05*	−0.05*	−0.06*	−0.04*	−0.34*	0.15*	−0.12*	−0.22*	−0.01	−0.21*	−0.23*	0.05*	−0.00	−0.07*	−0.09*	0.00	−0.11*	−0.26*	−0.37*	1.00	
(22)	0.00	0.02*	0.03*	−0.06*	−0.04*	0.08*	0.02*	−0.11*	0.31*	0.03*	0.05*	0.10*	0.01	0.02*	0.04*	0.08*	0.00	0.07*	0.29*	−0.40*	−0.35*	1.00

*Note.* (1) self-employment entry; (2) new marriage; (3) childbirth; (4) new divorce; (5) widowhood; (6) age; (7) working age; (8) gender; (9) married; (10) rural; (11) education; (12) job tenure; (13) health condition; (14) immigrant; (15) number of preschool-aged children; (16) number of school-aged children; (17) self-employment experience; (18) childcare expenses; (19) sole breadwinner; (20) primary breadwinner; (21) secondary breadwinner; (22) co-breadwinner.

* significant at 1% level.

### Unification Transitions: Marriage and Childbirth

Table 3 reports discrete-time logit estimates of self-employment entry, with demographic, household, industry, province, and wave controls, and standard errors clustered at the individual level.

**Table 3.** Self-Employment Entry as a Function of Life Events, Household Dynamics, and Caregiving Intensity.

		Marriage		Childbirth			Divorce			Widowhood		Co-occurrence
Variables	Baseline	(H1a)	(H1b)	(H2a)	(H2b)	(H2c)	(H3a)	(H3b)	(H3c)	(H4a)	(H4b)	(H5)
Life event												
Marriage		0.40** (0.137)										0.388** (0.147)
Childbirth				0.304* (0.134)								0.198 (0.141)
Divorce							0.313 (0.246)	−0.144 (0.324)	0.672 (0.433)			
Widowhood										−0.436* (0.187)	−0.114 (0.228)	
Co-occurring marriage and childbirth												1.544** (0.280)
Capability conditions												
Newly Married Women × Secondary Breadwinner			1.058* (0.458)									
Newly married women			0.77** (0.190)									
New mother × Sole / Primary breadwinner					−0.708* (0.302)							
New mother					0.475* (0.248)							
New Father × Caregiving Intensity						−0.328* (0.180)						
New father						0.83** (0.267)						
Newly Divorced Status × Childcare Expenses								1.81** (0.482)				
Newly Divorced Status × L2. Sole/Primary Breadwinner									−1.278* (0.620)			
Newly Widowed Status × Working Age											−0.769 (0.464)	
Working Age											0.180 (0.122)	
Household Dynamics												
Sole breadwinner	0.339** (0.099)	0.108 (0.079)	0.146* (0.079)	0.34** (0.100)		0.33* (0.100)	0.30* (0.100)	0.29** (0.100)		0.178* (0.081)	0.177* (0.081)	0.123 (0.080)
Primary breadwinner	−0.225** (0.071)	−0.219** (0.071)	−0.23** (0.071)	−0.23** (0.071)		−0.22** (0.071)	−0.21** (0.070)	−0.21** (0.070)		−0.23** (0.071)	−0.222** (0.071)	−0.220** (0.071)
Breadwinner (sole + primary)					−0.070 (0.066)				0.29** (0.082)			
Secondary breadwinner	0.422** (0.079)	0.417** (0.078)	0.38** (0.078)	0.43** (0.080)	0.36** (0.079)	0.43** (0.080)	0.45** (0.079)	0.45** (0.080)	0.46** (0.082)	0.38** (0.078)	0.382** (0.078)	0.491** (0.077)
Caregiving constraints												
Childcare expenses	−0.163 (0.103)	−0.158 (0.105)	−0.144 (0.104)	−0.147 (0.103)	−0.161 (0.105)	−0.161 (0.103)	−0.18* (0.105)	−0.187* (0.105)	−0.099 (0.100)	−0.097 (0.104)	−0.097 (0.104)	−0.117 (0.104)
Number of pre-school children	0.249** (0.055)	0.313** (0.055)	0.30** (0.054)	0.16** (0.067)	0.25** (0.060)	0.23** (0.063)	0.25** (0.057)	0.25** (0.057)	0.25** (0.057)	0.29** (0.054)	0.30** (0.054)	0.250** (0.069)
Number of school-aged children	0.153** (0.031)	0.189** (0.031)	0.18** (0.030)	0.16** (0.032)	0.15** (0.031)	0.16** (0.031)	0.16** (0.032)	0.15** (0.032)	0.14* (0.035)	0.17** (0.031)	0.17** (0.031)	0.194** (0.031)
Demographics												
Age	0.009* (0.002)	0.011** (0.002)	0.01** (0.002)	0.01** (0.002)	0.01** (0.002)	0.01** (0.002)	0.01** (0.002)	0.01** (0.002)	0.01** (0.002)	0.01** (0.002)	0.01** (0.002)	0.011** (0.002)
Gender	0.103* (0.057)	0.082 (0.057)	0.067 (0.057)	0.103* (0.057)	0.055 (0.057)	0.126* (0.058)	0.088 (0.057)	0.089 (0.057)	0.129* (0.063)	0.091 (0.057)	0.094 (0.057)	0.071 (0.057)
Married	0.298** (0.087)			0.28** (0.088)	0.029 (0.072)	0.28** (0.088)	0.32** (0.088)	0.32** (0.088)	0.34** (0.083)	0.29** (0.087)	0.29** (0.087)	
Immigrant	0.106 (0.066)	0.138* (0.066)	0.114* (0.066)	0.105 (0.066)	0.100 (0.066)	0.106 (0.066)	0.131* (0.066)	0.130* (0.066)	0.089 (0.074)	0.112 (0.066)	0.110 (0.066)	0.127* (0.066)
Rural	0.168** (0.064)	0.116* (0.064)	0.18** (0.064)	0.17** (0.064)	0.16** (0.064)	0.17** (0.064)	0.106 (0.064)	0.109* (0.064)	0.143* (0.070)	0.17** (0.064)	0.17** (0.064)	0.104 (0.063)
Education	0.187** (0.030)	0.143** (0.030)	0.20** (0.030)	0.19** (0.030)	0.19** (0.030)	0.19** (0.030)	0.13** (0.030)	0.13** (0.030)	0.14** (0.033)	0.19** (0.030)	0.19** (0.030)	0.138** (0.030)
Self-employment exp	3.105** (0.091)	3.117** (0.090)	3.11** (0.090)	3.11** (0.091)	3.11** (0.091)	3.10** (0.091)	3.12** (0.090)	3.12** (0.090)	3.64** (0.105)	3.10** (0.091)	3.11** (0.091)	3.121** (0.090)
Health condition	0.075 (0.047)	0.096* (0.048)	0.075 (0.046)	0.075 (0.047)	0.070 (0.047)	0.076 (0.047)	0.094* (0.048)	0.094* (0.048)	0.114* (0.053)	0.077 (0.046)	0.079* (0.046)	0.090* (0.048)
Job tenure	−0.016** (0.003)	−0.016** (0.003)	−0.02** (0.003)	−0.02** (0.003)	−0.02** (0.003)	−0.02** (0.003)	−0.02** (0.003)	−0.02** (0.003)	−0.01** (0.004)	−0.02** (0.003)	−0.02** (0.003)	−0.016** (0.003)
Industry (NAICS) Chi^2^(15)	740.8**	741**	748**	740**	741**	743**	732**	731**	705**	747**	719**	750**
Province Chi^2^(8)	35.55**	34.82**	36.7**	35.4**	38.5**	35.4**	33.1**	32.5**	27.2**	38**	38**	34**
Year Chi^2^(3/4)	53.22**	49.5**	51.6**	52.1**	54**	52.7**	51.1**	51.2**	86**	53**	53.2**	48.1**
Wald Chi^2^	3,400**	3,197**	3,433**	3,412**	3,409**	3,412**	3,055**	3,076**	2,828**	3,413**	3,440**	3,184**
*n*	81,794	69,336	69,336	81,794	81,794	81,794	64,869	64,869	53,233	69,856	69,856	64,874

*Note.* All NAICS, province, and yearly control variables are included in the analysis but omitted from the table for brevity (full results available upon request). Instead, Wald test statistics for the joint significance of the controls are reported.

* significant at 5% level; ** significant at 1% level.

#### Marriage (H1a–H1b)

Model H1a shows that marriage is positively associated with self-employment entry (β=.40,p<.01) , supporting H1a. Substantively, this corresponds to about 49% higher odds of entry during the interval for respondents who marry between waves $\left(e^{0.40}-1\right)$ . Interpreted through the household capability framework, marriage appears to expand buffering capacity, resource pooling, and shared risk-bearing, thereby increasing tolerance for earnings volatility and making entry more feasible for some household roles.

Model H1b sharpens this result by showing that marriage-linked feasibility is not evenly distributed within couples. The interaction for *newly married women* in *secondary-breadwinner* positions is positive and statistically significant (β=1.058,p<.05) , supporting H1b. This indicates that, for secondary-earning women, the association between marriage and self-employment entry is substantially stronger than implied by the relevant main effects alone. In other words, the marriage association is not simply a secondary-earner effect; it is filtered through gendered conversion conditions. Marriage-related buffering is more likely to translate into usable flexibility and a socially legible route into flexible work for women whose earnings are supplementary.

#### Childbirth (H2a–H2c)

Model H2a indicates that childbirth is positively associated with self-employment entry (β=.304,p<.05) , corresponding to about 36% higher odds of entry during the interval $\left(e^{0.304}-1\right)$ . This is consistent with the argument that early parenthood can increase the salience of flexibility and autonomy in work arrangements while reshaping household care and income constraints (Craig & Powell, 2012).

The estimates also show that this is not a uniform parenthood–entry association. For mothers, the pattern depends on whether childbirth coincides with income anchoring. In the model that distinguishes mothers and tests earning-role contingency under H2b, the interaction between new motherhood and sole- or primary-breadwinner status is negative and significant (β=−.708,p<.05) . In odds terms, this implies that, among new mothers, the association with entry is about 50% lower when the mother is the household’s sole or primary earner $\left(e^{-0.708}-1\right)$ . In capability terms, when intensive caregiving is combined with primary income responsibility, the household’s tolerance for earnings volatility tightens and time constraints become more binding. Under these conditions, childbirth is more likely to compress than expand the conversion capability required for self-employment.

A parallel but gender-asymmetric contingency emerges for fathers. In the father-specific model (H2c), new fatherhood is positively associated with entry (β=.83,p<.01) , but this association is significantly attenuated as caregiving intensity increases (β=−.328,p<.05) , supporting H2c. Each additional preschool-aged child corresponds to about a 28% reduction in fathers’ odds of entry $\left(e^{-0.328}-1\right)$ , offsetting part of the otherwise positive association. In this sense, self-employment entry after childbirth depends not on parenthood alone, but on gendered role alignment. Entry is lower for women when childbirth coincides with primary breadwinning, and lower for men when fatherhood coincides with intensive caregiving. In both cases, tighter time and income constraints reduce the capacity to absorb volatility, making self-employment less feasible. This pattern is consistent with household specialization and gendered career constraints, in which the reallocation of earning and caregiving responsibilities is the key mechanism (Lundberg & Pollak, 1996; Yavorsky et al., 2015).

### Dissolution Transitions: Divorce and Widowhood

In Model H3a, the divorce coefficient is positive but statistically insignificant (β=.313) , indicating no uniform average association between divorce and self-employment entry. This is consistent with our argument that the implications of marital dissolution depend on how financial, caregiving, and time-related responsibilities are reallocated across former partners.

Once these post-dissolution conditions are taken into account, a more structured pattern emerges. Contrary to H3b, the interaction between newly divorced status and childcare-expense responsibility is positive and statistically significant (β=1.81,p<.01) . This should not be interpreted to mean that childcare costs encourage entrepreneurship. Rather, it is consistent with a capability-constraint interpretation: when divorce coincides with non-discretionary childcare expenses and the loss of income pooling, some individuals may face a tighter time–liquidity squeeze, making self-employment a necessity-driven adjustment under constrained options rather than an opportunity-enabled choice.

Contrary to H3c, the interaction between newly divorced status and prior sole- or primary-breadwinner status is negative and statistically significant (β=−1.278,p<.05) . This indicates that divorce is especially constraining for those who carried the main household income role before separation. Prior work shows that primary earners often absorb a larger share of post-divorce costs, including housing, legal expenses, and child-related transfers, while also facing persistent wealth losses (Amato, 2010; Braver et al., 2011; Killewald, 2016). In capability terms, divorce shifts income from slack to obligation, making earnings volatility harder to absorb and self-employment less feasible. The constraint is therefore role-based rather than inherently gendered: whoever bears the main earning and childcare responsibilities faces the strongest post-separation pressure against entry.

Widowhood is associated with a lower likelihood of entering self-employment (β=−.436,p<.05) , supporting H4a and indicating that spousal loss erodes the capabilities needed to take on entrepreneurial risk. The loss of shared income, emotional support, and household risk pooling tightens constraints precisely when financial and logistical demands often increase (Moen, 2016; Tamborini et al., 2015). H4b is not supported: the widowhood × working-age interaction is not significant (β=−.769) . This suggests that the constraint does not vary systematically by remaining work horizon. Instead, the pattern points to a common constraint mechanism across age groups: the abrupt collapse of mutual support and planning capacity is difficult to offset through individual adjustment alone.

### Co-occurring Marriage and Childbirth: Rapid Family Formation

Experiencing marriage and childbirth within the same 2-year period is strongly and positively associated with self-employment entry (β=1.544,p<.01) , supporting H5. The association for co-occurrence is substantially larger than that of either transition on its own: marriage is positively related to entry (β=.388,p<.01) , whereas childbirth alone is not statistically significant (β=.198) . This points to more than a simple additive pattern. When partnership formation and the onset of intensive caregiving occur within the same interval, households must renegotiate time, income pooling, and risk sharing simultaneously. Under this compressed reorganization, self-employment may become a practical adjustment by offering scheduling control and autonomy that are harder to secure in standard wage work. The results therefore underscore that timing matters: entry is more likely when multiple household shifts occur together than when they unfold more gradually.

The controls are broadly consistent with a capability-based interpretation of entry. Age and education are positively associated with entry, while job tenure is negatively associated, suggesting that human capital supports entry but wage-employment embeddedness raises switching costs. Immigrants also show higher entry, consistent with self-employment serving as an alternative pathway when paid employment is constrained. Household earning roles also matter in a role-contingent way. Sole and secondary earners are more likely to enter self-employment, while primary breadwinners are less likely to do so because their earnings anchor household stability and make volatility harder to absorb. Rural residence and the presence of preschool- and school-aged children are also positively associated with entry, consistent with contexts in which wage employment is less available or less flexible.

## Discussion

This study advances entrepreneurship theory by reframing self-employment entry as a household-level adaptation to life-course transitions rather than a direct expression of individual entrepreneurial preference or opportunity pursuit. Integrating life-course theory, household bargaining, and the capability approach, it shows that major family transitions—marriage, childbirth, divorce, widowhood, and the co-occurrence of marriage and childbirth—are associated with entry patterns consistent with contingent reallocation of conversion capability. In doing so, the findings refine persistent assumptions in the self-employment literature and clarify why identical life events are linked to divergent entry responses across individuals and households.

A first contribution is to clarify how self-employment entry should be interpreted in entrepreneurship research. Dominant models often explain entry in terms of autonomy, non-pecuniary utility, or opportunity and necessity conditions (Benz & Frey, 2008; Parker, 2018). The findings here suggest that such interpretations are incomplete when family transitions reorganize household roles and constraints. Although the observational design cannot rule out correlation between life-course transitions and autonomy preferences or opportunity conditions, the results indicate that household capability reallocation provides an additional and underdeveloped lens for understanding self-employment entry. In this view, self-employment following marriage, childbirth, divorce, or co-occurring marriage and childbirth is shaped not only by stable entrepreneurial preferences but also by how households reallocate risk, care, and income responsibility under changing conditions. This matters because prior work often treats family context as a background correlate of entry, whereas the findings show that household reorganization is itself a core source of heterogeneity in self-employment transitions (Aldrich & Cliff, 2003; Carter et al., 2017; Welter, 2011). Marriage increases entry for secondary earners but not for primary earners; childbirth enables entry when households can buffer income volatility but suppresses it when caregiving coincides with income anchoring; and divorce and widowhood constrain entry when shared buffers collapse. Taken together, these patterns suggest that self-employment entry is often a relational labor-market adjustment embedded in household reorganization rather than an individual choice made in isolation.

A notable nuance to the first contribution concerns divorce. Contrary to the initial expectation, the effects of divorce appear to operate through capability compression rather than capability release. Although dissolution can in principle restore autonomy or intensify pressure to generate income (Tamborini et al., 2015), the results indicate that the loss of risk pooling and income buffering constrains entry among sole and primary earners. This suggests that post-divorce entry depends less on divorce itself than on how dissolution restructures earning responsibility, caregiving, and household buffering capacity. Self-employment following divorce therefore appears to reflect a constrained adjustment among those whose labor can still be reallocated, reinforcing the argument that adaptive entry is shaped by household capability allocation rather than by necessity alone. Unlike marriage and childbirth—where household roles are more visibly structured by gendered expectations—the constraining effects of divorce and widowhood appear to operate more directly through the loss of shared buffers and household support, producing similar capability compression among individuals in comparable roles.

A second contribution is to identify contingent capability reallocation as the household-level mechanism through which similar life events generate divergent entry responses. Rather than operating as uniform shocks, family transitions have consequences that depend on how households redistribute caregiving, flexibility, and earning responsibility. Marriage, childbirth, divorce, and widowhood therefore do not simply alter household resources; they reorganize who can convert time, support, and available means into a viable self-employment option. This extends family embeddedness research by showing that households are not merely contexts for entrepreneurial decision-making, but active allocators of conversion capability (Aldrich & Cliff, 2003; Carter et al., 2017). The mechanism is also structured by gendered role allocation. Consistent with gender-and-entrepreneurship research, gender matters here not as an intrinsic difference in entrepreneurial orientation, but as part of the relational process through which labor adjustability, caregiving, and earnings responsibility are distributed within households (Ahl & Marlow, 2012, 2021; Jennings & Brush, 2013). By shifting attention from individual motivation to household capability allocation, this study helps explain why similar life events produce different entry responses across individuals and why prior findings on marriage, childbirth, and dissolution often appear mixed rather than uniform.

A third contribution of this study is to show that the timing and compression of life events matter. When marriage and childbirth co-occur within the same observational interval, households face a compressed period of adjustment, and self-employment entry is significantly more likely than after either event alone. This extends life-course theory by showing that temporal proximity between transitions is itself consequential for occupational adjustment (Elder & Giele, 2009). For entrepreneurship research, it suggests that family context should be understood not only in terms of event type or household structure but also in terms of how tightly transitions unfold in time. From a capability perspective, the co-occurrence of marriage and childbirth narrows the time available for households to renegotiate labor allocation, build buffers, and absorb intensified care demands through marginal adjustments within wage employment. Under such conditions, reallocating one member’s labor toward self-employment becomes more feasible than would be expected from either transition in isolation. More broadly, this finding shows that household capability reallocation is not only event-specific but also temporally contingent.

In summary, these findings reposition self-employment entry as a relational outcome of household negotiation, not a uniform expression of entrepreneurial intent. Research on self-employment benefits from recognizing that entry often reflects institutionalized risk allocation as much as opportunity pursuit. Policies that assume self-employment is universally voluntary, such as generic credit access or training programs, may fail to address those whose capability is most constrained precisely when entry is most likely, including after household dissolution, during periods of intensified caregiving, or in income-dependent households. Interventions aligned with life-course timing and household constraints, such as portable benefits, childcare access for the self-employed, or transition support following family disruption, may therefore be more effective than generic self-employment promotion. Such measures do not promote self-employment per se; they reshape the capability conditions under which entry becomes sustainable and less precarious.

This study has several limitations that should inform interpretation and future research. First, although the longitudinal design, lagged covariates, and controls for prior self-employment reduce concerns about simultaneity, the analysis cannot fully eliminate endogeneity arising from unobserved traits (e.g. risk tolerance or autonomy orientation) or anticipatory behavior around family transitions. The results should therefore be interpreted as patterned associations rather than causal effects. The contribution lies in showing that self-employment entry varies systematically with the reallocation of household capability, even when causal attribution remains limited. Second, household roles and constraints are measured from the respondent’s perspective. The analysis therefore captures how risk, care, and income responsibility are reallocated to the individual who adjusts their labor-market position, not the joint employment behavior of all household members. While this fits the paper’s focus on who absorbs adjustment after life-course transitions, future research using linked partner data could observe household-level allocation processes more directly. Third, the analysis focuses on entry rather than post-entry persistence or performance. Capability reallocation may facilitate entry while constraining sustainability, particularly after dissolution or bereavement. Finally, other life-course shocks, such as illness, disability, or elder care, may generate different forms of capability compression or expansion and warrant further study. Examining how these non-familial shocks interact with household structure across institutional contexts would further extend the capability-based perspective advanced here.

In conclusion, this study shows that self-employment entry following major life-course transitions is often shaped by contingent reallocation of conversion capability within households. By treating households as active allocators of risk, care, and income responsibility, it offers a relational account of self-employment entry that sharpens theoretical interpretation and policy relevance.

## Figures and Tables

**Figure A1.**
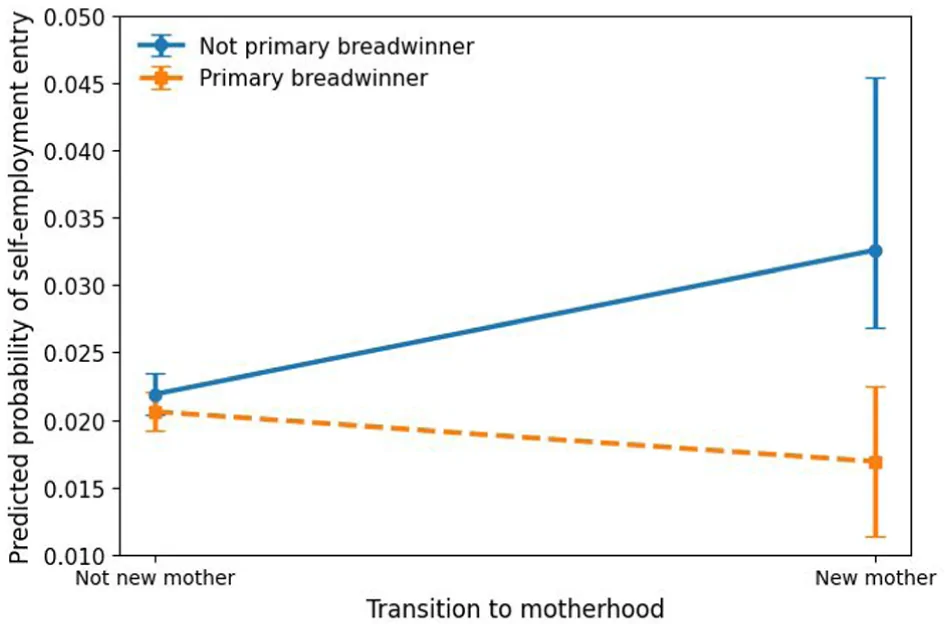
Predictive margins of motherhood and breadwinner status. *Note.* New motherhood increases self-employment entry among non-primary breadwinners but reduces entry among primary breadwinners.

**Figure A2.**
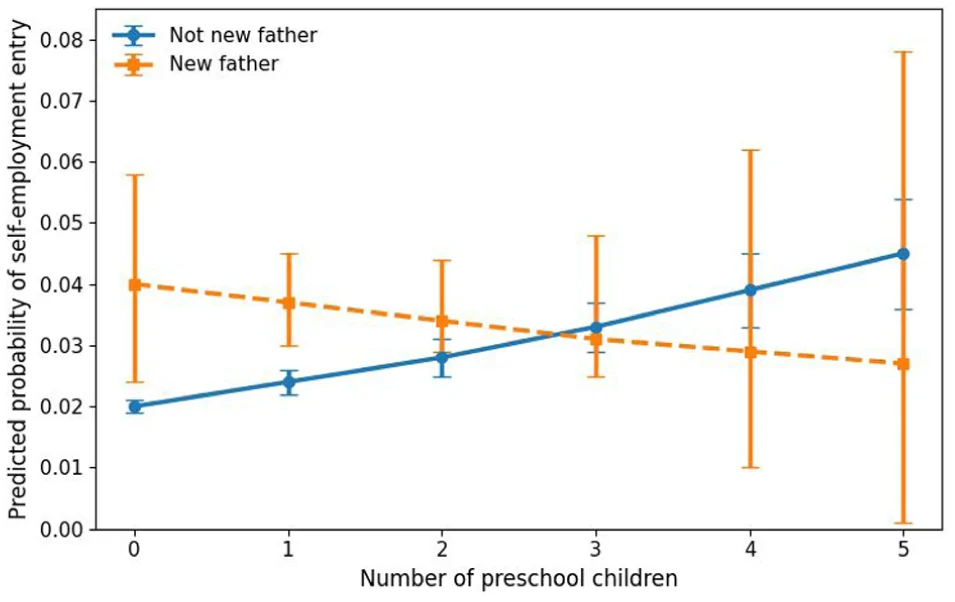
Interaction of new fatherhood and the number of preschool children. *Note.* Self-employment entry increases with caregiving intensity among non-new fathers but declines among new fathers.

**Figure A3.**
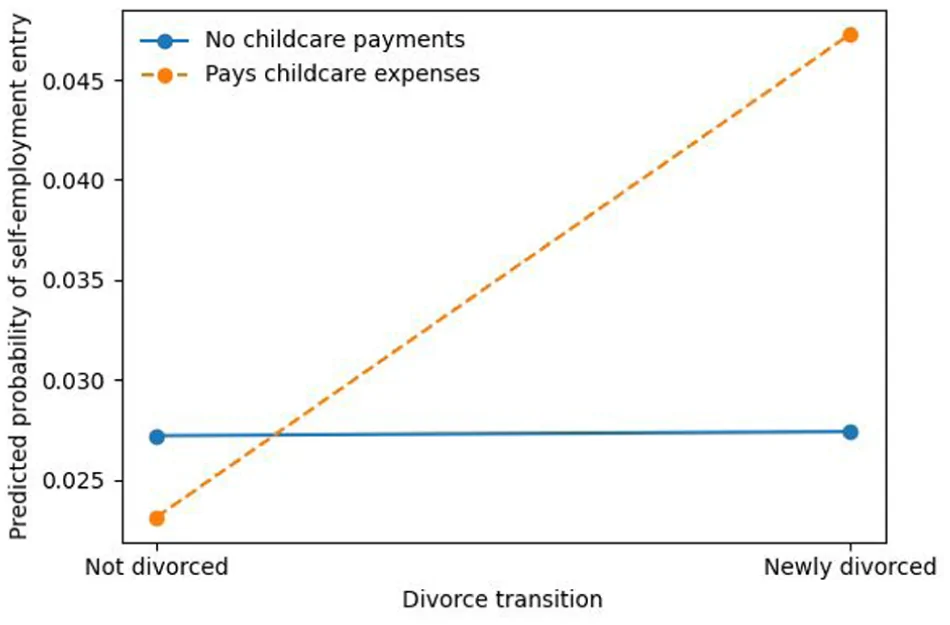
Interaction of divorce and childcare expenses on self-employment entry. *Note.* Divorce is associated with increased self-employment entry only among individuals responsible for childcare expenses.

**Figure A4.**
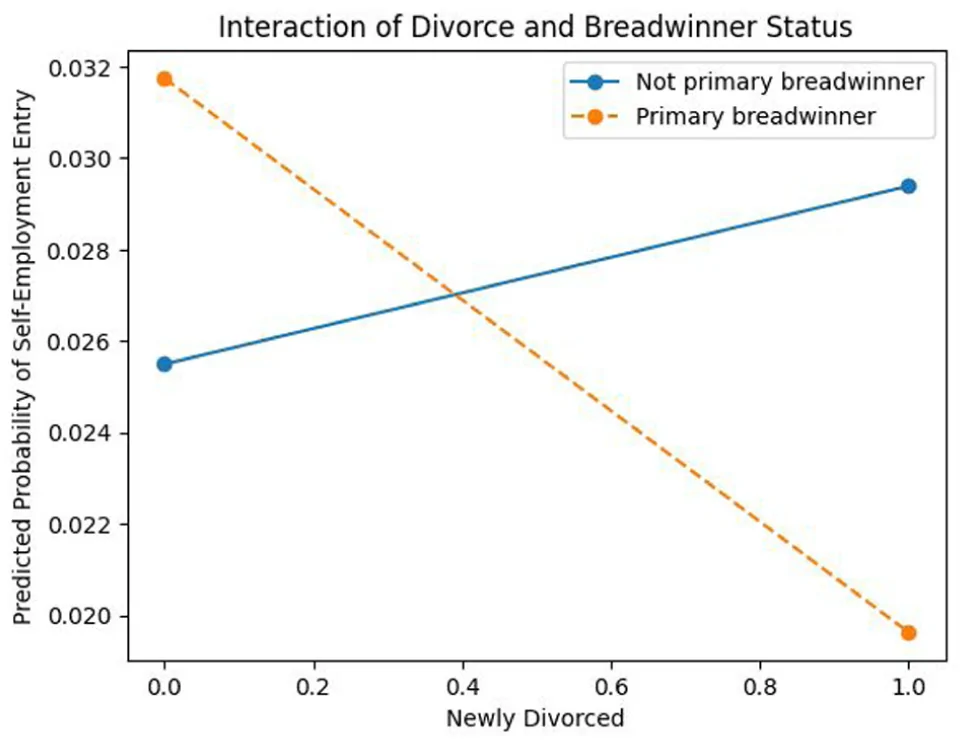
Interaction of divorce and prior breadwinner on self-employment entry. *Note.* Divorce increases entry among individuals who were not primary earners prior to divorce but suppresses entry among former primary earners.

**Figure A5.**
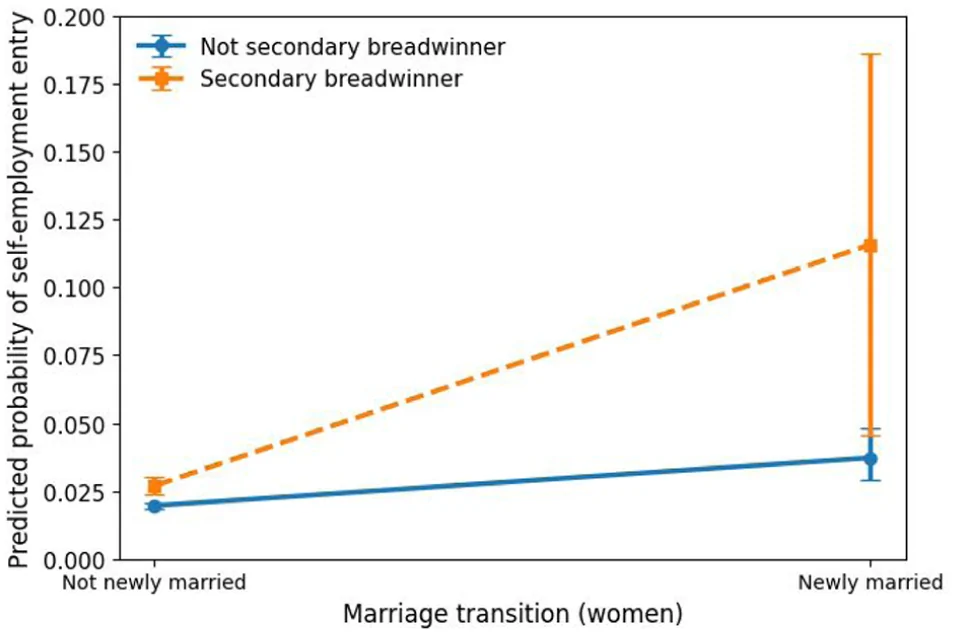
Interaction of marriage and secondary breadwinner status among women. *Note.* Marriage is associated with a substantial increase in self-employment entry among women who were secondary breadwinners prior to marriage, while the effect is modest among non-secondary breadwinners.

**Figure A6.**
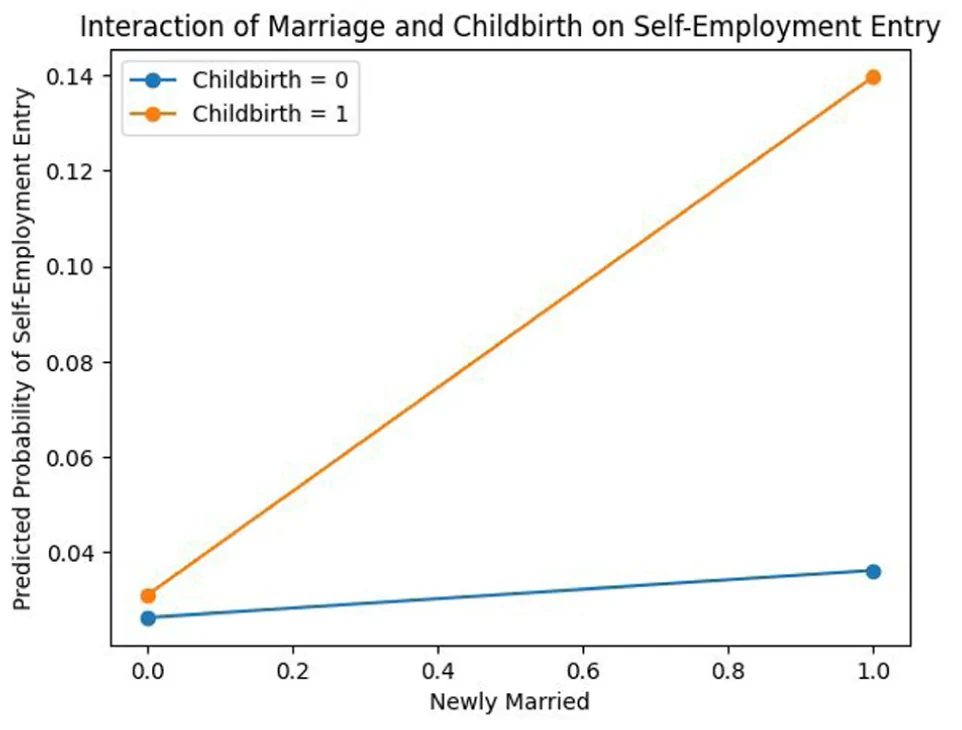
Interaction of marriage and childbirth on self-employment entry. *Note.* Joint occurrence of marriage and childbirth is associated with a substantially higher probability of entry than either event in isolation.

## Appendix A

Author's calculations based on discrete-time event-history (logit) models using Statistics Canada's Longitudinal and International Study of Adults (LISA), 2012–2020.

## Notes

1. Marriage, divorce, and widowhood were observed across four waves (2014–2020) by tracking changes in marital status; the first wave (2012) is excluded because prior status cannot be determined. Childbirth is observed across five waves (2012–2020) using respondents’ direct reports (“Did you give birth to a child since the last survey?”), cross-validated with the reported birth year of the youngest child.
